# Reabilitação funcional para pacientes acometidos por covid-19

**DOI:** 10.15649/cuidarte.2276

**Published:** 2021-09-21

**Authors:** Júlio César de Souza, Joel Saraiva Ferreira, Geize Rocha Macedo de Souza

**Affiliations:** 1 Secretaria Municipal de Saúde, Rua Sergipe, Sul, Brasil. E-mail: jcsouza13@hotmail.com Secretaria Municipal de Saúde, Rua Sergipe Sul Brasil jcsouza13@hotmail.com; 2 Universidade Federal de Mato Grosso do Sul, Brasil. E-mail: joel.ferreira@ufms.br Universidade Federal de Mato Grosso do Sul Universidade Federal de Mato Grosso do Sul Brazil joel.ferreira@ufms.br; 3 Secretaria Municipal de Saúde, Sul, Brasil. E-mail: geize01@yahoo.com.br

O vírus SARS-CoV-2 foi detectado na cidade de Wuhan, na China, no final de 2019(1). Desde então, a alta transmissibilidade deste novo coronavirus e a elevada morbimortalidade relacionada à COVID-19, a infecção viral causada pelo SARS-CoV-2, cresceu exponencialmente em todo o planeta, culminando, primeiramente, em uma emergência de saúde pública de importância internacional e, logo em seguida, em uma grave pandemia.(2),(3)

A transmissão do vírus ocorre tanto por vias respiratórias quanto extrarrespiratórias e durante o período de desenvolvimento da COVID-19 no organismo humano a apresentação clínica inclui sintomas relacionados a alterações no olfato, paladar e complicações gastrointestinais, além do acometimento de forma mais acentuada do sistema respiratório.(4)

Em consequência dos sintomas da COVID-19, muitos pacientes precisam ser internados e, em alguns casos, são submetidos a procedimentos invasivos para garantir a condição fisiológica básica para a manutenção da vida. Durante o período de internação os pacientes desenvolvem condição clínica com debilitação fisiológica e a aptidão física geral reduz sensivelmente.(5) Ao receber alta hospitalar, muitos desses pacientes precisam de tratamentos voltados à recuperação da condição física debilitada pela ação da doença.(6)

A Secretaria Municipal de Saúde Pública (SESAU) de Campo Grande/MS, na Região Centro-Oeste do Brasil, se mostrou atenta a esta demanda. O Serviço de Doenças Crônicas Não Transmissíveis (DANT), juntamente com a Coordenadoria de Atenção Especializada, estruturou no Sistema Único de Saúde (SUS) um serviço de reabilitação funcional destinado às pessoas com doenças crônicas não transmissíveis, que estiveram internadas por infecção pelo SARS-CoV-2 e precisam de atenção especializada para restabelecer a capacidade de trabalho e de realização das atividades da vida diária.

O atendimento é realizado na Unidade Especializada de Reabilitação e Diagnóstico (UERD), que faz parte da Rede Municipal de Saúde (REMUS). Um projeto de reabilitação funcional foi elaborado pela equipe técnica da SESAU, para ser desenvolvido por meio de uma equipe multiprofissional formada por fisioterapeuta, profissional de educação física, nutricionista e psicólogo. As intervenções são realizadas com o uso de Projetos Terapêuticos Singulares (PTS), seguindo critérios de inclusão e exclusão pré-estabelecidos(7), para pacientes com doenças crônicas não transmissíveis, em condição de pós-internação decorrente de COVID-19. O encaminhamento dos pacientes, vindos dos demais pontos da REMUS, é realizado por médicos dos três níveis de atenção à saúde (primária, secundário e terciário), via Sistema de Regulação (SISREG).

Foi estabelecido um fluxo para as intervenções, sendo que no primeiro atendimento cada paciente passa por um acolhimento dado pela equipe multiprofissional (fisioterapeuta, profissional de educação física, nutricionista e psicólogo) em uma consulta compartilhada. Nesta etapa a equipe faz uma triagem e identifica a condição clínica da pessoa atendida.

Posteriormente, os profissionais constroem, de forma coletiva, um PTS. Nos dias subsequentes, o paciente é contatado para iniciar o tratamento de reabilitação, por meio de agendamento nos dias e horários em que haja compatibilidade entre as vagas ofertadas e a disponibilidade do paciente.

O protocolo do PTS consiste de intervenções com frequência de duas vezes por semana, com alternância entre os profissionais que desenvolvem as atividades. Os pacientes são atendidos, inicialmente, com um total de 10 sessões de 40 a 60 minutos cada, durante cinco semanas. Se a equipe identificar que há indicação de continuidade do tratamento, o paciente pode ser mantido no programa por mais sessões, com avaliação caso a caso.

O processo de alta do paciente ocorre quando são finalizadas as sessões do PTS. Esse processo é caracterizado pelas orientações ao paciente e sua família/cuidador, com encaminhamento profissional para a contrarreferência da unidade de saúde à qual o paciente está vinculado na atenção primária, por meio de relatório de alta, com informações referentes ao tratamento e orientações, visando a integralidade do cuidado. Com isso, a atenção primária dará prosseguimento do cuidado em saúde ao usuário em seu território de abrangência.

O programa de reabilitação funcional prevê uma abordagem multiprofissional, com longitudinalidade do cuidado e respeito às singularidades de cada paciente. Os propósitos são aliviar os efeitos das disfunções musculares e respiratórias, mitigar os danos do imobilismo após longo período de internação hospitalar, além de atenuar as sequelas psicológicas e necessidades nutricionais.

Para obter a almejada melhora da saúde geral e da qualidade de vida das pessoas atendidas, cada área profissional envolvida no PTS intervém da seguinte forma:

A Fisioterapia atua em duas vertentes: a) fisioterapia respiratória, com o objetivo de aumentar a capacidade pulmonar e recuperar disfunções referentes ao processo de respiração; b) fisioterapia motora, com o objetivo de corrigir e restabelecer as condições físicas do paciente.

A abordagem do profissional de Educação Física é voltada à melhora dos componentes cardiorrespiratório e neuromuscular, visando a promoção da autonomia funcional, o controle dos fatores de risco para comorbidades, correção postural e manutenção do peso ideal. O atendimento inclui o treinamento aeróbio, o treinamento resistido, ações de educação em saúde, além de incentivo à continuidade da prática de atividades físicas após o término das sessões do PTS, para manutenção e ampliação dos ganhos obtidos.

A Nutrição atua com o propósito de manutenção do bom estado do sistema imunológico dos pacientes e, para isso, realiza avaliação nutricional e orientações ligadas à promoção de uma alimentação saudável.

A Psicologia disponibiliza suporte psicológico, com oferecimento de um espaço para que o paciente compartilhe seus sentimentos e necessidades, buscando a auto segurança. Com isso, objetiva-se minimizar o trauma psicológico decorrentes do período de internação, que pode ter gerado reações emocionais como medo, angústia, ansiedade e tristeza.

O conjunto de intervenções propostas representa uma readequação do serviço de saúde, em função da demanda oriunda da pandemia de COVID-19. Para isso, são necessárias estratégias capazes de proporcionar recuperação físico-funcional e reintegração social dos indivíduos infectados pelo vírus SARS-CoV-2, uma vez que essa infecção pode prejudicar a capacidade de realizar atividades da vida diária e a funcionalidade dos indivíduos que passaram por internação hospitalar, além de alterar o desempenho profissional e dificultar a interação social, com possibilidade de aumento de fatores de risco para doenças crônicas não transmissíveis.(8)

Nesse sentido, o PTS realizado por uma equipe multiprofissional, como no programa relatado, é um dispositivo de cuidado que visa a resolutividade dos casos complexos por meio da corresponsabilização e utilização dos recursos de todos os envolvidos, por meio do desenvolvimento de ações terapêuticas a partir de uma reflexão sistematizada. Para isso, o PTS deve ser abordado em duas perspectivas, uma delas centrada na clínica ampliada e outra na recuperação da autonomia da vida afetiva do sujeito, tal como preconizado no SUS.(9) Em ambas as perspectivas sempre são considerados o sujeito, as equipes de saúde, o território e a família onde o PTS é desenvolvido.(10)

A utilização do PTS no âmbito da saúde pública melhora não só o processo de trabalho como o vínculo entre todos os envolvidos - profissionais de saúde e pacientes, potencializando assim a assistência integral à saúde na produção do cuidado, em função da troca de saberes no âmbito multiprofissional.(11)-(13)

Nota-se, portanto, que o processo de tratamento dos pacientes com COVID-19 é fundamental não apenas durante a fase hospitalar, mas também após alta melhorada. Dentre as estratégias disponíveis para atender os pacientes na rede pública de saúde, o desenvolvimento de programas de reabilitação funcional, por meio de PTS e intervenções multiprofissionais, pode garantir a continuidade do acompanhamento, promovendo a recuperação de implicações provocadas pela doença, proporcionando mais qualidade de vida às pessoas no período pós- internação.
